# Effectivity of Saffron Extract (Saffr’Activ) on Treatment for Children and Adolescents with Attention Deficit/Hyperactivity Disorder (ADHD): A Clinical Effectivity Study

**DOI:** 10.3390/nu14194046

**Published:** 2022-09-28

**Authors:** Hilario Blasco-Fontecilla, Esther Moyano-Ramírez, Olga Méndez-González, María Rodrigo-Yanguas, Marina Martin-Moratinos, Marcos Bella-Fernández

**Affiliations:** 1 Hospital Universitario Puerta de Hierro Majadahonda, 28222 Majadahonda, Spain; 2 Faculty of Medicine, Universidad Autónoma de Madrid, 28029 Madrid, Spain; 3 CIBERSAM (Centro de Investigación en Salud Mental), Carlos III Institute of Health, 28029 Madrid, Spain; 4 ITA Mental Health, 28043 Madrid, Spain; 5 Faculty of Psychology, Universidad Autónoma de Madrid, 28049 Madrid, Spain; 6 Department of Psychology, Universidad Pontificia de Comillas, 28049 Madrid, Spain

**Keywords:** ADHD, saffron, CPT

## Abstract

Attention Deficit/Hyperactivity Disorder is the most prevalent neurodevelopmental disorder worldwide. Choice treatment includes psychostimulants, but parents tend to be reluctant to administer them due to side effects, and alternatives are needed. Saffron extract is a natural stimulant that has been proven safe and effective for treating a variety of mental disorders. This study compares the efficacy of saffron and the usual treatment with methylphenidate, using objective and pen-and-paper tests. We performed a non-randomized clinical trial with two groups, methylphenidate (*n* = 27) and saffron (*n* = 36), in children and adolescents aged 7 to 17. Results show that the efficacy of saffron is comparable to that of methylphenidate. Saffron is more effective for treating hyperactivity symptoms, while methylphenidate is more effective for inattention symptoms.

## 1. Introduction

Attention Deficit/Hyperactivity Disorder (ADHD) is one of the most prevalent neurodevelopmental disorders among children, adolescents, and adults worldwide [1]. ADHD symptoms typically encompass hyperactivity, inattention, and impulsivity. Multimodal treatment, including pharmacotherapy, psychotherapy, and psychoeducation, is the treatment of choice for ADHD [2]. Pharmacotherapy consists of stimulants (i.e., methylphenidate, lisdexamphetamine) or non-stimulant drugs (i.e., atomoxetine, guanfacine). Although ADHD medications are safe, and the profile of side effects is usually mild, many parents and patients are reluctant to take these medications [3]. Accordingly, different authors have advocated for the use of alternative treatments such as neurofeedback [4], serious video games [5,6], and/or nutritional supplements [7,8].

Within nutritional supplements, saffron has recently been postulated as one of the most interesting alternative treatments for ADHD. Saffron is a spice extracted from saffron crocus (crocus sativus) that has traditionally been used as an additive and food colorant worldwide, but also as a natural remedy for several diseases given its anti-inflammatory and antioxidant properties. Accordingly, saffron has been used to treat chronic diseases such as rheumatoid arthritis, inflammatory bowel diseases, Alzheimer’s, and several cancers (e.g., colon, stomach, breast, lung, and skin) [9]. Moreover, recent evidence also suggests that saffron may improve the lipid profile and help to modulate hypertension [10]. Furthermore, there is increasing evidence that saffron, probably due to some key constituents (namely, crocin, picrocrocin, and safranal) exerts protection of the central nervous system [11]. Indeed, saffron has several psychoactive properties [12,13,14], and acts on NDMA and GABA receptors [15], facilitating dopamine, serotonin, and noradrenaline secretion [16]. Given that, first, both dopamine and noradrenaline are the core neurotransmitters associated with ADHD, and second, saffron extract is a safe, natural substance [13,17,18,19], thus alleviating parental fears regarding the stimulant-drug based treatment of ADHD, it seems plausible to test whether saffron has some potential to treat ADHD. However, there is only limited literature on the potential therapeutic use of saffron for ADHD.

Recently, three clinical trials tested saffron’s efficacy to treat ADHD symptoms in adolescents and adults [20,21,22]. All three of them showed, in general terms, the efficacy of saffron. Baziar et al. [20] did not find significant differences between treatment with methylphenidate and saffron, whereas Khaksarian et al. [21] found an added effect of saffron and methylphenidate compared to the effect of methylphenidate alone. Pazoki et al. [22] reported that a combination of methylphenidate and saffron better improved ADHD symptoms compared with methylphenidate alone. Unfortunately, none of these trials used objective measures for ADHD symptoms; rather, they used pen-and-paper tests. Another limitation of the previous studies is that none of them measured executive functions. This is relevant because approximately 50% of children and adolescents with ADHD have executive dysfunction [23]. Furthermore, to our knowledge, there is no previous literature specifically testing the potential use of saffron in the treatment of executive dysfunction. Finally, children with ADHD frequently suffer from sleep disturbances and daytime sleepiness [24]. Given that saffron improves sleep quality, latency, and duration [25], saffron may address both issues by improving both ADHD core symptoms and sleep. 

In this paper, we present a non-randomized study using objective measures, alongside a battery of subjective, psychometric scales (see Methods below), in order to measure both core ADHD symptoms and executive functions. We compared the efficacy of Saffr’activ^®^ (a specific trademark saffron commercialized in Spain by Massó; https://www.cqmasso.com/, accessed on 12 August 2022) compared to methylphenidate in core ADHD symptoms and executive functions. Our main hypothesis is that saffron presents similar efficacy compared with methylphenidate. We based the main hypothesis on the above-mentioned studies reporting positive results about the efficacy of saffron in different ADHD populations. We operationalized this hypothesis into two more specific hypotheses. First, patients taking saffron and methylphenidate show similar improvements in objective measures for core ADHD symptoms (inattention and hyperactivity); and second, patients taking saffron and methylphenidate show similarly improved executive functioning. The second hypothesis may help to elucidate whether or not the improvements in ADHD symptoms, in the event that we do find such findings, are explained by improvements in executive functions. 

## 2. Materials and Methods

### 2.1. Design

A single-center, prospective, naturalistic, non-randomized, non-blind, pre–post intervention study was conducted in the Child and Adolescent Mental Health Services (CAMHS) at Puerta de Hierro University Hospital in Majadahonda, Spain. After a thorough explanation, written reported consent was signed by the patients and their parents or legal tutors.

### 2.2. Sample

Participants were recruited from outpatients aged 7 years old or above, who met the criteria for ADHD diagnosis following the Diagnostical and Statistical Manual (DSM-5), confirmed by a child psychiatrist. For 20% difference between treatments, 80% statistical power, and 2.5% statistical significance, the sample size was calculated as 35 participants per group. All the patients were naïve (they had never received pharmacological treatment for ADHD or had not received it in the six months prior to starting the trial).

### 2.3. Interventions

The sample was divided into two groups: Group 1 received psychoeducation and extended-release methylphenidate (tritiated up to a dose of 1 mg/kg per day), while Group 2 received psychoeducation and saffron (30 mg/day). The patients and their parents could choose their treatment group after a full explanation of the study and the properties of both methylphenidate and saffron. For each participant, the treatment duration was 3 months. Group 1 received one dose of extended-release methylphenidate in the morning (before/after meal), whereas patients of group 2 were recommended to take one dose of saffron in the evening (before/after meal). We based this recommendation on the well-known properties of saffron in improving sleep quality, latency, and duration [25]. Measurements (see below) were taken at the beginning and at the end of the treatment duration.

### 2.4. Measures

Similar to previous studies [20,21], we used raw scores of several subjective measures. Moreover, we also used a performance-based task to objectively measure executive function. All of them are described in this section.

The severity of ADHD was measured using two scales. First, the SNAP-IV, Spanish version [26] was used. The SNAP-IV is an 18-item questionnaire that measures two of the core ADHD symptoms, inattention and hyperactivity. The 18-item checklist is scored on a 4-point Likert scale ranging between Not At All (0) and Very Much (3) (range: 0 to 54). The SNAP-IV is one of the most frequently used questionnaires to evaluate the response to treatment. Thus, the SNAP-IV was used in the NIMH Collaborative Multisite Multimodal Treatment (MTA) study of children with ADHD [27]. Second, we also used the Conners’ Parent Rating Scale Revised (CPRS-R) [28], a 10-item, 4-point Likert screening test designed for assessing ADHD symptoms. This instrument was constructed by reducing the number of items from longer versions of the Conners scales. It evaluates 10 behavioral statements rated on a 4-point Likert scale (range: 0–30) [29]. The cut-off point for a screening diagnosis of ADHD is 15 or higher [30]. 

Furthermore, we evaluated executive functions using the Behavioral Rating Inventory of Executive Function—Second Edition (BRIEF-2) [31], Spanish version [32]. The BRIEF-2 consists of 72 items that measure eight domains of executive function: inhibition, flexibility, emotional control, initiative, working memory, planification, self-supervision, material organization, and task supervision. This test shows adequate psychometric properties. The BRIEF-2 has demonstrated good reliability (internal and test–retest) and satisfactory to good convergent and construct validity in different countries, including Spain [33,34] 

Finally, sleep quality was measured with the Sleep Disturbance Scale for Children (SDSC) [35]. The SDSC is 26-item test measuring sleep problems. The test has six factors, but we focused on items related to initiating and maintaining sleep. SDSC has adequate psychometric factors for children and adolescents from 5 to 15 years old, and is usually considered the benchmark questionnaire for evaluating sleep in children and adolescents [36]. 

In addition to these subjective measures, we used the Conners’ Continuous Performance Test, version 3 (CPT-3) [37]. The CPT-3 is a task that assesses impulsivity and sustained attention. The CPT-3 is an objective measure that, unlike pen-and-paper tests, as in the other measures previously described, does not depend on the subject’s or an external observer’s impression. The former version shows adequate internal consistency and test–retest reliability (except the omissions measure), but a low correlation with subjective measures for executive functions [38].

### 2.5. Statistical Analyses

First, Kolmogorov–Smirnov tests were performed to assess the normality assumption for the variables. For most variables, normality could not be assumed. Thus, we employed non-parametric tests when possible. Mann–Whitney tests were performed to test the main effects of group (methylphenidate vs. saffron) and time (pre–post) factors. Two-way 2 × 2 ANOVAs were performed with time (pre vs. post treatment) and group (stimulant vs. saffron) as factors and each measure as a dependent variable, to test the interactions between these two factors. Statistical analyses were performed in SPSS v. 26.0.

## 3. Results

Most patients (32 out of 36 in the saffron arm and 24 out of 27 in the methylphenidate arm) completed the study. Furthermore, 7 patients from the methylphenidate arm (25.9%) and 10 patients from the saffron arm (31.2%) reported side effects (Fischer’s exact test: *p* = 0.552).

Table 1 shows the sociodemographic variables for the two experimental groups at the beginning of the trial.

Table 2 shows the baseline characteristics in clinical variables and test scores for the two groups. The results are similar to those reported in the literature. For instance, the BRIEF-2 scores are fairly similar to those reported in a study comparing ADHD and non-ADHD children and adolescents aged 5 to 18. Thus, patients diagnosed with inattention had a global executive composite of 68.54 [39]. 

Table 3 shows the results from the two-way ANOVA analyses. To assess the differential efficacy of saffron and methylphenidate, the most important analysis is the interaction between factors. In this sense, results show that differential effects do not reach statistical significance. Moreover, regardless of the treatment, patients showed improvements in most subjective pen-and-paper tests (except the Hyperactivity subscale of SNAP-IV) and two measures of the CPT.

**Figure 1:**
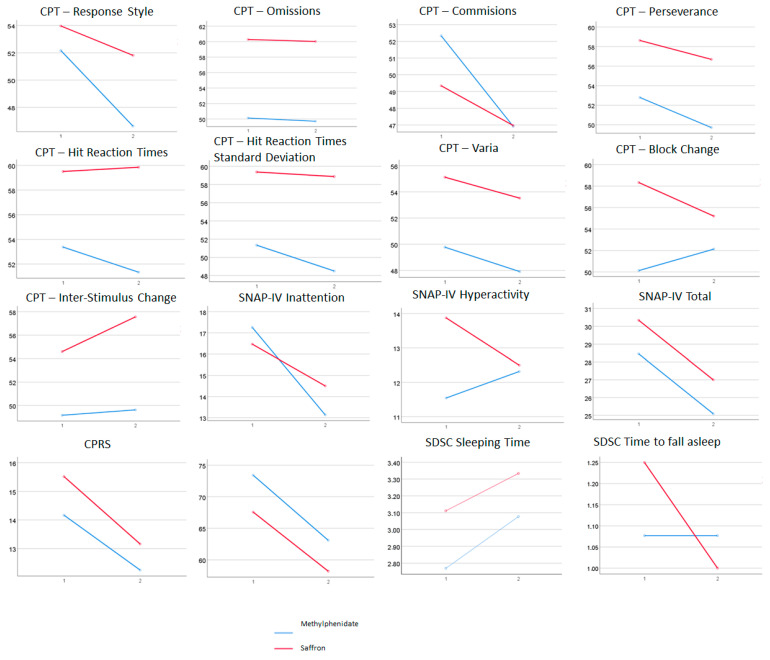
Interaction graphs for the ANOVAs. SDSC scales are not expressed in sleep hours or minutes until falling asleep, but in scores from Likert-type scales. Mean scores of SDSC Sleeping Time are approximately equivalent to sleep for 7–8 h (pre) and 8–9 h (post). Mean scores of SDSC Time to Fall Asleep are approximately equivalent to around 30 min (pre in saffron group), 15 (post in saffron group), and 15–30 min (pre and post in methylphenidate group).

We observed a global effect for treatment in most subjective measures. CPT measures showed significant treatment effects only for response style and number of commission errors. The absence of significant effects for interactions suggests that both treatments had similar benefits in the patients included in this study. However, in Figure 1, we can observe, although not reaching statistical significance, differential effects for treatment (both methylphenidate and saffron improve ADHD symptoms) and effect–group interactions (in some cases, one of the two treatments has larger effects).

In Figure 1, we can see the interactions between pre–post and treatment group, regardless of their significance. It is noteworthy that for the SNAP-IV inattention subscale, the methylphenidate group improved more than the saffron group, and the opposite effect was observable in the hyperactivity subscale. Moreover, in the CPT Block Change measure, the improvement was slightly better in the saffron group, while, for Commissions, the improvement was better in the methylphenidate group. Interestingly, patients taking saffron showed improvements in the time to fall asleep, unlike patients with methylphenidate. Furthermore, we also observed a pronounced decreased in the time to fall asleep only in the saffron arm (statistically non-significant). 

Table 4 shows the comparisons between groups at the end of the treatment. For several CP measures, results are better for the methylphenidate group compared with the saffron group. None of these differences reach statistical significance.

## 4. Discussion

The present pilot study compares the efficacy of saffron and methylphenidate in children and adolescents diagnosed with ADHD. The most relevant finding is that both treatments showed statistically significant improvements in both core ADHD symptoms and executive functions. More importantly, they were comparable in terms of efficacy, measured with both pen-and-paper tests filled out by parents, and objective measures (CPT-3). It is also noteworthy that both treatments were well tolerated, and no significant side effects were reported. Finally, a pronounced difference was found in the saffron arm, in the sense that the parents of the children treated with saffron reported a pronounced improvement in the time to fall asleep. 

Consistent with our hypothesis, we found a comparable improvement in the mean vs. the baseline score in both parent-rated, subjective scales and the CPT-3 in children either using methylphenidate or saffron. This is in keeping with the recent literature. Several recent clinical trials examined the efficacy and safety of saffron extract to treat ADHD symptoms [20,21,22]. All three of them found that saffron is an effective and safe treatment for ADHD. Baziar et al. [20] showed a similar effect for saffron and methylphenidate in children. Khaksarian et al. [21] showed that combining saffron with methylphenidate was more effective for treating ADHD symptoms in children than methylphenidate alone. Pazoki et al. [22] found a similar boosting effect in adults. However, a common limitation of these three studies is the usage of pen-and-paper tests to assess efficacy. In this study, we compared the efficacy of saffron and methylphenidate through a battery of subjective and objective tests. On the other hand, although the three studies mentioned above did not use objective measures, they were methodologically sounder that ours, as the three studies were randomized, double-blind clinical trials. 

We found that saffron and methylphenidate were both comparably effective for treating ADHD symptoms in children, as pointed out by the non-significant interaction effects from the ANOVAs. Nonetheless, the graphical interpretation of the ANOVAs showed interesting trends: while methylphenidate tended to be more effective for inattention, saffron tended to be more effective for hyperactivity, as measured by the SNAP-IV scale. Additionally, another interesting finding was that it not only improved ADHD core symptoms, but also executive functions. Furthermore, the improvement in the executive functions was moderate, compared with the mild improvements in core ADHD symptoms. Executive functions include some cognitive processes (i.e., inhibition, working memory, multi-tasking, monitoring of actions) that are core to ADHD [40]. It is possible that the improvement in ADHD measures is at least in part due to the improvement in executive functions. There is a wide range of literature supporting the improvement in executive functions in children with ADHD treated with methylphenidate [41]. Unfortunately, we did not find any single study testing the potential improvement in executive functions using saffron.

Furthermore, our results from SDSC suggest that, although not reaching statistical significance, saffron has an impact on improving time to fall asleep, which methylphenidate does not have. Both groups improve sleeping time similarly, which is usually an issue when treating ADHD patients. This finding is clinically relevant given the well-known association between ADHD and sleep problems [42]. Furthermore, our findings are in keeping with the extensive literature demonstrating that saffron can improve sleep quality. Thus, a recent randomized, double-blind, controlled study using objective measures demonstrated that six weeks of saffron supplementation led to (1) increased time in bed assessed by actigraphy; (2) improved ease of falling sleep; and (3) improved sleep quality, latency, and duration [25]. Moreover, the combined improvement in core ADHD symptoms, executive functions, and sleep quality may be explained by saffron’s properties. Saffron is rich in two carotenoids (crocins and crocetin), pirocrocin, and safranal, which have powerful antioxidant, anti-tumor, and anti-inflammatory properties that may explain the emerging literature testing its role in neuropsychiatric and neurodegenerative diseases [14].

Regarding the CPT-3, patients with saffron improved most in Block Change measures, a measure related to sustained attention, while patients with methylphenidate improved most in Commissions, a parameter more closely related to impulsivity. Interestingly, none of the samples improved in measures of Inter-Stimulus Interval changes, another measure related to sustained attention. These results contrast those obtained by Khaksarian et al. [21], who found that adding saffron to a methylphenidate-based treatment yielded improved benefits in both hyperactivity and inattention. This difference can nonetheless be explained by the different design (they compared a combination of saffron and methylphenidate against methylphenidate, while we compared saffron alone with methylphenidate).

The discrepancy between objective and subjective measures for the hyperactivity–impulsivity domain can be explained in two ways: first, objective and subjective measures tend to show low correlations [38,43]; and second, the initial difference between groups in scores for the Hyperactivity subscale from SNAP-IV was larger than the initial difference for Commissions in CPT.

Although our results are in line with previous trials, these results have to be interpreted taking into account several limitations. First, unlike previous studies in this field, this pilot study was neither randomized nor blind; rather, patients were able to choose between treatments and the PI was aware of their selection. This limitation makes our study more sensitive to selection bias. However, as displayed in Table 1, both subgroups were fairly comparable in terms of severity. Furthermore, parents were blind to baseline assessments. Moreover, some authors have emphasized that the expectation of benefit does not influence the treatment response in children with ADHD [44]. Second, we did not use interviews, such as the Diagnostic Interview Schedule for Children, version IV (DISC-IV), to fully corroborate the clinical diagnosis of ADHD. However, ADHD is a clinical diagnosis, and both the CPRS-R and SNAP-IV are ecologically valid measures of children’s behavior at home [45]. Furthermore, the children who were treated with methylphenidate used different commercially available products that had mild differences regarding bioavailability. Finally, the sample size was relatively small, but it was fair for the saffron group. However, even if the methylphenidate group was smaller than desirable—perhaps suggesting that parents preferred to choose a natural treatment over standard pharmacological treatment—the rate of subject loss in both groups was very low (4 children from 36 in the saffron arm, and 3 children from 26 in the methylphenidate arm). In any case, further studies with larger samples are warranted. 

On the other hand, the most relevant strengths of the present study are, first, the use of objective measures, and second, that the improvements in both groups included both ADHD core symptoms and executive functions, and both were observed using subjective and objective measures, thus giving consistency to our study. 

## 5. Conclusions

In conclusion, this pilot study presents evidence of the efficacy and safety of saffron extract compared to methylphenidate in a sample of children and adolescents with ADHD. Although comparable in general terms, saffron tends to be more effective for hyperactivity symptoms, while methylphenidate is more effective for inattention symptoms. Furthermore, both treatments improved the number of sleeping hours, but only saffron made it easier to fall asleep.

## Figures and Tables

**Table 1:** Sociodemographic factors *.

	Saffron Group (*n* = 36)	Methylphenidate Group (*n* = 27)	Comparison Contrast (Mann–Whitney’s U or Fisher’s Exact Test)	*p* Value
Age	10.57 (3.22)	11.74 (3.335)	U = 383.5	0.203
Gender				
Male	25 (69.4%)	17 (63%)	N/A	0.602
Female	11 (30.6%)	10 (37%)		
Nationality				
Spanish	35 (97.2%)	26 (96.3%)	N/A	0.670
Russian	1 (2.8%)	1 (3.7%)		
Previous psychological treatment				
Yes	9 (25%)	7 (19.4%)	N/A	0.554
No	27 (75%)	20 (80.6%)		
Familial psychiatric history				
Yes	27 (75%)	19 (70.4%)	N/A	0.370
No	9 (25%)	8 (29.6%)		

* Quantitative variables are described through mean and standard deviation and compared through Mann–Whitney tests (assumptions for *t*-tests are not met). Categorical variables are described through absolute frequencies and percentages and compared through Fisher’s exact tests (assumptions for chi-square tests are not met).

**Table 2:** Clinical variables and test scores **.

	Saffron Group (*n* = 36)	Methylphenidate Group (*n* = 27)	Comparison Contrast (Mann–Whitney’s U or Fisher’s Exact Test)	*p* Value
SNAP-IV				
Inattention	16.81 (5.382)	16.93 (6.288)	U = 474	0.867
Hyperactivity	13.86 (6.766)	11.15 (6.66)	U = 375.5	0.124
Total	30.67 (9.78)	28.07 (10.366)	U = 419.5	0.355
CPRS-10	15.34 (6.087)	14.48 (5.80)	U = 441	0.654
BRIEF-2	67.86 (21.354)	69.46 (20.44)	U = 458	0.887
CPT-3				
Response Style	52.00 (11.130)	52.68 (7.958)	U = 495	0.903
Omissions	58.42 (17.868)	52.04 (11.286)	U = 451	0.472
Commissions	48.03 (8.531)	51.71 (7.102)	U = 370	0.069
Perseverance	57.42 (15.030)	54.29 (12.915)	U = 467.5	0.620
Hit RT	60.67 (13.076)	54.5 (9.403)	U = 358	0.048
Standard Deviation Hit RT	58.56 (16.804)	53.32 (12.798)	U = 430.5	0.320
Variability	54.56 (14.773)	51.29 (11.476)	U = 398	0.458
HRT Block change	55.77 (12.478)	49.29 (13.302)	U = 335	0.032
HRT ISI	55.08 (13.731)	49.75 (9.819)	U = 424	0.278

Abbreviations: Hit RT (Hit Reaction time); HRT Block Change (Hit Reaction Time Block Change); HRT ISI (Hit Reaction Time Inter-Stimulus Interval). ** Quantitative variables are described through mean and standard deviation and compared through Mann–Whitney tests (assumptions for *t*-tests are not met). Categorical variables are described through absolute frequencies and percentages and compared through Fisher’s exact tests (assumptions for chi-square tests are not met).

**Table 3:** Two-way ANOVA results.

	Group Effect		Treatment Effect		Interaction Effect (Group × Treatment)	
	F Statistics	*p* Value	F Statistics	*p* Value	F Statistics	*p* Value
SNAP-IV						
Inattention	0.053	0.820	16.577	<0.001	2.082	0.155
Hyperactivity	0.666	0.418	0.163	0.688	2.069	0.156
Total	0.660	0.420	6.960	0.011	<0.001	0.994
CPRS-10	0.596	0.443	11.754	0.001	0.124	0.726
BRIEF-2	1.071	0.306	16.096	<0.001	0.038	0.847
CPT-3						
Response Style	2.304	0.135	12.914	0.001	2.498	0.120
Omissions	7.556	0.008	0.025	0.874	0.002	0.968
Commissions	0.587	0.447	13.992	<0.001	2.132	0.150
Perseverance	5.344	0.025	2.270	0.138	0.118	0.732
Hit RT	6.381	<0.001	0.672	0.416	1.327	0.254
Standard Deviation Hit RT	7.034	0.010	0.900	0.347	0.441	0.509
Variability	3.391	0.072	1.042	0.313	0.006	0.938
HRT Block Change	6.046	<0.001	0.091	0.764	1.862	0.178
HRT ISI	5.077	0.028	0.731	0.396	0.392	0.534
SD						
Sleep Hours	1.150	0.292	1.623	0.213	0.042	0.839
Time to Fall Asleep	0.020	0.888	0.481	0.493	0.481	0.493

Abbreviations: Hit RT (Hit Reaction time); HRT Block Change (Hit Reaction Time Block Change); HRT ISI (Hit Reaction Time Inter-Stimulus Interval). SDSC (Sleep Disturbance Scale for Children).

**Table 4:** Post-treatment differences.

	Saffron Group (*n* = 32)	Methylphenidate Group (*n* = 24)	Mann–Whitney’s U	*p* Value
SNAP-IV				
Inattention	−1.97 (5.21)	−4.13 (5.83)	279.5	0.130
Hyperactivity	−1.37 (5.62)	0.77 (5.03)	298	0.340
Total	−3.34 (8.91)	−3.36 (9.57)	337.5	0.798
CPRS-10	−2.35 (4.40)	−1.92 (4.81)	350.5	0.714
BRIEF-2	−9.37 (17.50)	−10.32 (17.45)	316.5	0.802
CPT-3				
Response Style	−2.16 (8.55)	−5.54 (7.01)	284	0.097
Omissions	−0.25 (17.42)	−0.42 (12.35)	324.5	0.323
Commissions	−2.37 (6.84)	−5.42 (8.75)	303	0.179
Perseverance	−1.94 (12.58)	−3.08 (12.02)	347	0.539
Hit RT	0.34 (8.86)	−2.04 (5.68)	335	0.416
Standard Deviation Hit RT	−0.50 (14.89)	−2.83 (9.92)	333.5	0.403
Variability	−1.60 (13.29)	−1.86 (9.32)	255	0.669
HRT Block Change	−3.13 (13.36)	2.00 (14.19)	278.5	0.156
HRT ISI	2.97 (16.61)	0.46 (12.07)	327	0.345
SD				
Sleep Hours	−0.22 (0.65)	−0.31 (1.60)	107	0.668
Time to Fall Asleep	−0.25 (0.97)	0.00 (1.08)	97.5	0.206

## Data Availability

The data presented in this study are available on request from the corresponding author. The data are not publicly available due to confidentiality issues.

## References

[1] Polanczyk G., De Lima M.S., Horta B.L., Biederman J., Rohde L.A. (2007). The worldwide prevalence of ADHD: A systematic review and metaregression analysis. Am. J. Psychiatry.

[2] Dalrymple R.A., Maxwell L.M., Russell S., Duthie J. (2020). NICE guideline review: Attention deficit hyperactivity disorder: Diagnosis and management (NG87). Arch. Dis. Child. Educ. Pract..

[3] Toomey S.L., Sox C.M., Rusinak D., Finkelstein J.A. (2012). Why do children with ADHD discontinue their medication?. Clin. Pediatrics.

[4] Purper-Ouakil D., Blasco-Fontecilla H., Ros T., Acquaviva E., Banaschewski T., Baumeister S., Bousquet E., Bussalb A., Delhaye M., Delorme R. (2021). Personalized at-home neurofeedback compared to long-acting methylphenidate in children with ADHD: NEWROFEED, a European randomized noninferiority trial. J. Child Psychol. Psychiatry.

[5] Rodrigo-Yanguas M., Martin-Moratinos M., Menendez-Garcia A., Gonzalez-Tardon C., Sanchez-Sanchez F., Royuela A., Blasco-Fontecilla H. (2021). A virtual reality serious videogame versus online chess augmentation in patients with attention deficit hyperactivity disorder: A randomized clinical trial. Games Health J..

[6] Rodrigo-Yanguas M., González-Tardón C., Bella-Fernández M., Blasco-Fontecilla H. (2022). Serious Video Games: Angels or Demons in Patients With Attention-Deficit Hyperactivity Disorder? A Quasi-Systematic Review. Front. Psychiatry.

[7] Pedersen P., Bjerrum M., Larsen P., Bjerrum S., Pedersen J., Peters M. (2017). Nutritional interventions to reduce symptoms in children and adults with attention deficit hyperactivity disorder: A scoping review protocol. JBI Evid. Synth..

[8] Granero R., Pardo-Garrido A., Carpio-Toro I.L., Ramírez-Coronel A.A., Martínez-Suárez P.C., Reivan-Ortiz G.G. (2021). The role of iron and zinc in the treatment of adhd among children and adolescents: A systematic review of randomized clinical trials. Nutrients.

[9] Ashktorab H., Soleimani A., Singh G., Amin A., Tabtabaei S., Latella G., Stein U., Akhondzadeh S., Solanki N., Gondré-Lewis M.C. (2019). Saffron: The golden spice with therapeutic properties on digestive diseases. Nutrients.

[10] Ghaffari S., Roshanravan N. (2019). Saffron; An updated review on biological properties with special focus on cardiovascular effects. Biomed. Pharmacother..

[11] Skladnev N.V., Johnstone D.M. (2017). Neuroprotective properties of dietary saffron: More than just a chemical scavenger?. Neural Regen. Res..

[12] Moradi K., Akhondzadeh S. (2021). Psychotropic Effects of Saffron: A Brief Evidence-based Overview of the Interaction Between a Persian Herb and Mental Health. J. Iran. Med. Counc..

[13] Avgerinos K.I., Vrysis C., Chaitidis N., Kolotsiou K., Myserlis P.G., Kapogiannis D. (2020). Effects of saffron (*Crocus sativus* L.) on cognitive function. A systematic review of RCTs. Neurol. Sci..

[14] El Midaoui A., Ghzaiel I., Vervandier-Fasseur D., Ksila M., Zarrouk A., Nury T., Khallouki F., El Hessni A., Ibrahimi S.O., Latruffe N. (2022). Saffron (*Crocus sativus* L.): A source of nutrients for health and for the treatment of neuropsychiatric and age-related diseases. Nutrients.

[15] Berger F., Hensel A., Nieber K. (2011). Saffron extract and trans-crocetin inhibit glutamatergic synaptic transmission in rat cortical brain slices. Neuroscience.

[16] Hosseinzadeh H., Karimi G., Niapoor M. Antidepressant effect of *Crocus sativus* L. stigma extracts and their constituents, crocin and safranal, in mice. Proceedings of the I International Symposium on Saffron Biology and Biotechnology 650.

[17] Broadhead G., Chang A., Grigg J., McCluskey P. (2016). Efficacy and safety of saffron supplementation: Current clinical findings. Crit. Rev. Food Sci. Nutr..

[18] Modaghegh M.-H., Shahabian M., Esmaeili H.-A., Rajbai O., Hosseinzadeh H. (2008). Safety evaluation of saffron (*Crocus sativus*) tablets in healthy volunteers. Phytomedicine.

[19] Mousavi B., Bathaie S.Z., Fadai F., Ashtari Z. (2015). Safety evaluation of saffron stigma (*Crocus sativus* L.) aqueous extract and crocin in patients with schizophrenia. Avicenna J. Phytomed..

[20] Baziar S., Aqamolaei A., Khadem E., Mortazavi S.H., Naderi S., Sahebolzamani E., Mortezaei A., Jalilevand S., Mohammadi M.-R., Shahmirzadi M. (2019). *Crocus sativus* L. versus methylphenidate in treatment of children with attention-deficit/hyperactivity disorder: A randomized, double-blind pilot study. J. Child Adolesc. Psychopharmacol..

[21] Khaksarian M., Ahangari N., Masjedi-Arani A., Mirr I., Jafari H., Kordian S., Nooripour R., Hassanvandi S. (2021). A Comparison of Methylphenidate (MPH) and Combined Methylphenidate with *Crocus sativus* (Saffron) in the Treatment of Children and Adolescents with ADHD: A Randomized, Double-Blind, Parallel-Group, Clinical Trial. Iran. J. Psychiatry Behav. Sci..

[22] Pazoki B., Zandi N., Assaf Z., Moghaddam H.S., Zeinoddini A., Mohammadi M.R., Akhondzadeh S. (2022). Efficacy and safety of saffron as adjunctive therapy in adults with attention-deficit/hyperactivity disorder: A randomized, double-blind, placebo-controlled clinical trial. Adv. Integr. Med..

[23] Lambek R., Tannock R., Dalsgaard S., Trillingsgaard A., Damm D., Thomsen P.H. (2011). Executive dysfunction in school-age children with ADHD. J. Atten. Disord..

[24] Becker S.P. (2020). ADHD and sleep: Recent advances and future directions. Curr. Opin. Psychol..

[25] Pachikian B.D., Copine S., Suchareau M., Deldicque L. (2021). Effects of saffron extract on sleep quality: A randomized double-blind controlled clinical trial. Nutrients.

[26] Grañana N., Richaudeau A., Gorriti C.R., O’Flaherty M., Scotti M.E., Sixto L., Allegri R., Fejerman N. (2011). Evaluación de déficit de atención con hiperactividad: La escala SNAP IV adaptada a la Argentina. Rev. Panam. Salud Pública.

[27] Group M.C. (2004). National Institute of Mental Health Multimodal Treatment Study of ADHD follow-up: 24-month outcomes of treatment strategies for attention-deficit/hyperactivity disorder. Pediatrics.

[28] Conners C.K., Sitarenios G., Parker J.D., Epstein J.N. (1998). The revised Conners’ Parent Rating Scale (CPRS-R): Factor structure, reliability, and criterion validity. J. Abnorm. Child Psychol..

[29] Conners C.K. (1990). Conners’ Abbreviated Symptom Questionnaire.

[30] Ullmann R.K., Sleator E.K., Sprague R.L. (1985). A change of mind: The Conners abbreviated rating scales reconsidered. J. Abnorm. Child Psychol..

[31] Gioia G.A., Isquith P.K., Guy S.C., Kenworthy L. (2015). BRIEF: Behavior Rating Inventory of Executive Function.

[32] Maldonado Belmonte M.J., Fournier del Castillo M.C., Martínez Arias R., Gioia G.A. (2017). BRIEF2: Evaluación Conductual de la Función Ejecutiva.

[33] Parhoon K., Moradi A., Alizadeh H., Parhoon H., Sadaphal D.P., Coolidge F.L. (2022). Psychometric properties of the behavior rating inventory of executive function, (BRIEF2) in a sample of children with ADHD in Iran. Child Neuropsychol..

[34] Jiménez A., Lucas-Molina B. (2019). Dimensional structure and measurement invariance of the BRIEF-2 across gender in a socially vulnerable sample of primary school-aged children. Child Neuropsychol..

[35] Bruni O., Ottaviano S., Guidetti V., Romoli M., Innocenzi M., Cortesi F., Giannotti F. (1996). The Sleep Disturbance Scale for Children (SDSC) Construct ion and validation of an instrument to evaluate sleep disturbances in childhood and adolescence. J. Sleep Res..

[36] Mancini V.O., Pearcy B.T. (2021). Sensitivity of the child behaviour checklist sleep items and convergent validity with the Sleep Disorders Scale for Children in a paediatric ADHD sample. Sleep Med. X.

[37] Conners C.K. (2014). Conners CPT-3: Manual.

[38] Shaked D., Faulkner L.M., Tolle K., Wendell C.R., Waldstein S.R., Spencer R.J. (2020). Reliability and validity of the Conners’ continuous performance test. Appl. Neuropsychol. Adult.

[39] Jacobson L.A., Pritchard A.E., Koriakin T.A., Jones K.E., Mahone E.M. (2020). Initial examination of the BRIEF2 in clinically referred children with and without ADHD symptoms. J. Attent. Disord..

[40] Chan R.C., Shum D., Toulopoulou T., Chen E.Y. (2008). Assessment of executive functions: Review of instruments and identification of critical issues. Arch. Clin. Neuropsychol..

[41] Wu C.-S., Shang C.-Y., Lin H.-Y., Gau S.S.-F. (2021). Differential treatment effects of methylphenidate and atomoxetine on executive functions in children with attention-deficit/hyperactivity disorder. J. Child Adolesc. Psychopharmacol..

[42] Silvestri R. (2022). Sleep and ADHD: A complex and bidirectional relationship. Sleep Med. Rev..

[43] Spencer R., Drag L., Walker S., Bieliauskas L. (2010). Self-reports of cognitive function are not predictive of neuropsychological test performance among returning combat veterans. J. Rehabil. Res. Dev..

[44] Hazell P., Lewin T., Sly K. (2005). What is a clinically important level of improvement in symptoms of attention-deficit/hyperactivity disorder?. Aust. N. Z. J. Psychiatry.

[45] Sengupta S.M., Grizenko N., Thakur G.A., Bellingham J., DeGuzman R., Robinson S., TerStepanian M., Poloskia A., Shaheen S., Fortier M.-E. (2012). Differential association between the norepinephrine transporter gene and ADHD: Role of sex and subtype. J. Psychiatry Neurosci..

